# Evaluating the Precision of 3D Morphological Reconstruction for Asphalt Pavement Textures via Close-Range Photogrammetry

**DOI:** 10.3390/ma19183931

**Published:** 2026-09-16

**Authors:** Liang Song, Di Yun, Le Wang, Shasha Jiang, Jie Gao

**Affiliations:** 1 School of Traffic and Transportation Engineering, Xinjiang University, Urumqi 830017, China; slpl@sohu.com; 2 Xinjiang Uygur Autonomous Region Highway Development Center, Urumqi 830000, China; 3 School of Automobile and Traffic Engineering, Wuhan University of Science and Technology, Wuhan 430081, China; 4 Xinjiang Transportation Investment Group Construction Management Co., Ltd., Urumqi 830006, China; wangle_19840808@163.com (L.W.); 15099152901@163.com (S.J.); 5 School of Civil Engineering and Architecture, East China Jiaotong University, Nanchang 330013, China; gaojie@ecjtu.edu.cn

**Keywords:** close-range photogrammetry, 3D texture, asphalt pavement, accuracy, testing parameters

## Abstract

**Highlights:**

• Dense-graded mixtures outperform open-graded ones in reconstruction fidelity.
• Optimal photo counts are 17–25 for balancing accuracy and computational cost.
• Texture indicators correlate with laser scanning but show systematic offsets.
• Ssk and Sku are noise-sensitive and excessive photos may introduce instability.

**Abstract:**

Accurate 3D reconstruction of pavement textures is vital for assessing surface performance. This study systematically evaluates the factors influencing the precision of close-range photogrammetry for pavement texture characterization. Four typical mixtures, AC-13, AC-16, SMA-16, and OGFC-16, were reconstructed from 17 to 145 photographs, with high-precision laser scanning as the reference. Reconstruction quality was quantified by point cloud density, elevation deviations, and four texture parameters. Findings reveal that dense-graded yields 81–87% of points within 0–0.5 mm deviation, while open-graded achieves only 51%. Point cloud density increases with photo count but follows diminishing returns, stabilizing between 21 and 73 images. Photogrammetric texture parameters systematically underestimate laser values, yet preserve relative ranking among gradations. Ssk and Sku are highly sensitive to extreme noise, especially on fine-textured surfaces. These insights offer practical guidelines for implementing photogrammetric techniques in pavement engineering.

## 1. Introduction

Pavement texture directly influences the indentation deformation behavior of tires and the distribution of the actual contact area, and also plays a critical role in key performance indicators such as skid resistance, rolling resistance, and service durability, even for energy harvesting [1,2]. Consequently, accurately acquiring and rationally characterizing pavement surface texture features is a fundamental prerequisite for revealing tire-pavement contact mechanisms and optimizing pavement texture design [3,4].

Currently, various technologies have been employed for 3D pavement texture reconstruction, existing measurement technologies are categorized into two primary groups based on whether they maintain direct contact with the pavement surface: contact-based and non-contact measurement methods [5,6]. The most representative of the former is the contact surface profilometer, which reconstructs the pavement texture profile by recording elevation data as a micro-stylus traverses the measured surface [7]. However, this method is constrained by a limited measurement range, for example, the micro-stylus is incapable of detecting microtexture smaller than the stylus tip diameter. Furthermore, this approach is susceptible to wear or damage during the measurement process, which has restricted its widespread application [8].

Non-contact measurement methods, on the other hands, including industrial Computed Tomography (CT) [9,10], nano- or micro-CT [10], laser scanning, structured light scanning, binocular vision, and close-range photogrammetry [11,12,13]. While mobile or airborne LiDAR systems integrated with GPS/INS or total stations are typically deployed for larger-scale, corridor-level pavement surveys, their spatial resolution is generally insufficient for detailed micro-texture analysis at the aggregate scale [14]. Among these, industrial CT provides detailed internal structural information but is limited to small-scale laboratory specimens. Laser scanning provides accurate coordinate data of the specimen texture, offering high reliability for pavement texture reconstruction. Specifically, laser focus tracking methods can achieve an elevation precision of 0.1 μm when measuring pavement microtexture, although their application is largely confined to laboratory environment [15]. With the continuous advancement of laser scanning technology, numerous scholars have utilized various laser scanners to acquire authentic pavement textures and employed reverse reconstruction techniques for surface morphology restoration [16]. By calculating diverse texture indicators—such as arithmetic mean height and mean curvature—researchers have further analyzed the correlations between these metrics and various pavement performance characteristics [17,18]. Although laser scanning is a highly feasible detection method with moderate costs and robust anti-interference capabilities, suitable for both laboratory and field testing, the sharp trade-off between measurement precision and operational efficiency remains a significant challenge to resolve.

Computer vision-based pavement morphology measurement methods leverage image acquisition systems and digital image processing techniques to achieve 3D structural reconstruction through approaches such as photometric stereo and binocular stereo vision [9,10,11,12,13,14,15,16,17,18,19,20,21]. These methods offer several advantages, including equipment simplicity, cost-effectiveness, and operational ease, enabling large-scale detection of pavement structures in a non-invasive manner, demonstrating the practical viability of photogrammetric approaches for pavement texture assessment. However, these studies did not systematically investigate image acquisition quantities affect the geometric fidelity of 3D reconstruction. In recent years, vision-based reconstruction methods integrating deep learning and multi-scale image fusion technologies have continuously emerged, effectively enhancing the stability and precision of texture information extraction under complex conditions. While notable reconstruction accuracy by integrating transfer learning with PatchmatchNet-based multi-view stereo was achieved, the framework was primarily oriented toward texture depth estimation rather than systematic evaluation of acquisition parameter effects [22]. However, structured light scanning requires stringent ambient lighting conditions, while binocular vision systems involve complex calibration and their accuracy is restricted by baseline length [23,24]. Moreover, the interaction between image quantity and gradation-dependent reconstruction performance was not explored.

In contrast, close-range photogrammetry has gained considerable attention for 3D pavement texture reconstruction due to its cost-effectiveness, operational simplicity, and broad applicability [25,26]. However, existing research has primarily focused on algorithmic optimization and application validation, resulting in a scarcity of systematic studies regarding the factors influencing reconstruction accuracy [27,28,29]. The surface texture of porous or open-graded mixtures, such as porous ultra-thin asphalt overlays, poses additional challenges for 3D reconstruction due to their complex void structures and shadowing effects [30]. However, critical issues, such as the determination of optimal capturing parameters and the impact of diverse pavement types on reconstruction precision, remain insufficiently addressed.

Collectively, while these studies have advanced individual aspects of vision-based pavement morphology measurement, a systematic, multi-factor experimental framework that simultaneously accounts for the effects of pavement gradation type, image acquisition quantity, and their interactions on 3D reconstruction accuracy is still needed. To bridge this gap, this study selected four typical asphalt mixtures for investigation. High-precision laser scanning was first employed to establish a benchmark for texture reconstruction. Subsequently, video sequences of the specimen surfaces were captured, from which frames were extracted at equal intervals for photogrammetry-based reconstruction. The Iterative Closest Point (ICP) algorithm was utilized to analyze point cloud deviations, point cloud density, and deviations in 2D texture indicators. This research systematically evaluates the effects of gradation type and image quantity on reconstruction performance, providing a theoretical foundation for the engineering application of close-range photogrammetry in pavement texture detection.

## 2. Materials and Methods

### 2.1. Principles of Photogrammetry

The fundamental principle of close-range photogrammetry for 3D reconstruction is based on binocular or multi-view stereopsis. By capturing a sequence of overlapping images from different perspectives, the spatial coordinates of surface points can be mathematically determined through triangulation. All image sequences were ultimately imported into the 3DF Zephyr software 8.0 platform for 3D point cloud reconstruction and texture mapping.

**Figure 1:**
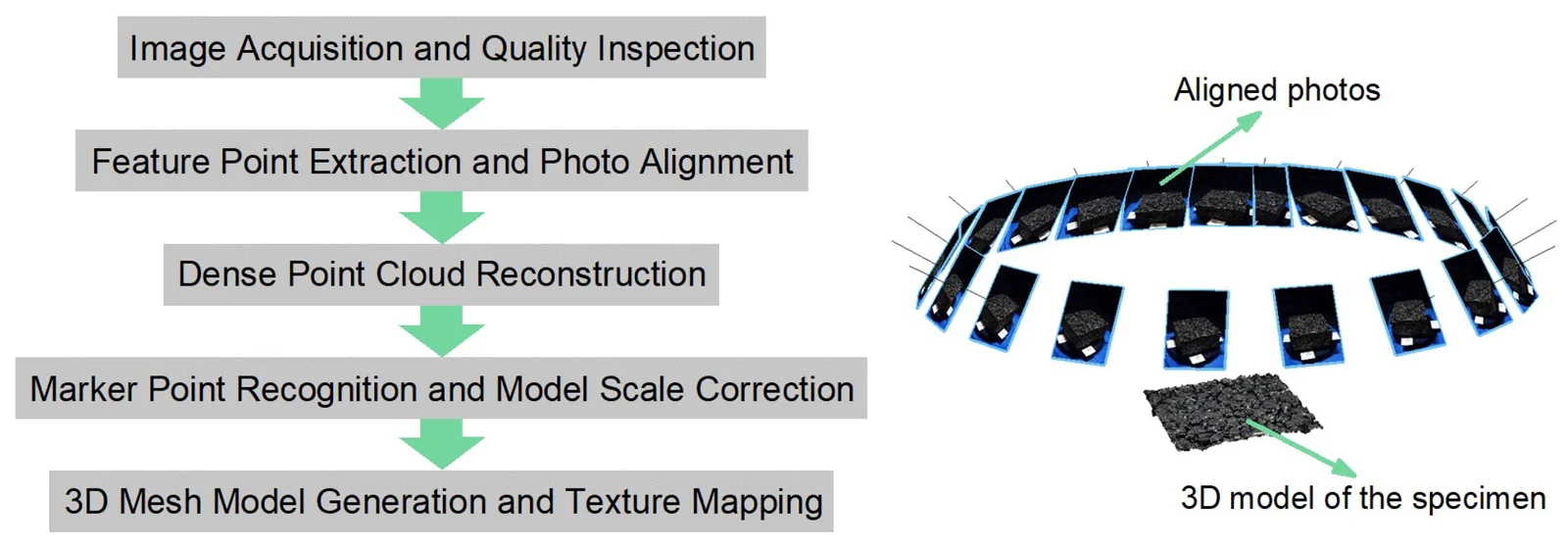
Workflow of close-range photogrammetric modeling.

The reconstruction process typically involves several critical stages (Figure 1): initial image acquisition and quality filtering to eliminate blurred, overexposed, or underexposed frames and ensure an image overlap of 60–80%; feature alignment utilizing the Scale-Invariant Feature Transform (SIFT) to extract feature points and RANSAC algorithms and bundle adjustment techniques to resolve camera poses, generating a sparse point cloud and completing the spatial alignment of the photographs; high-density reconstruction via Multi-View Stereo (MVS) matching techniques for depth estimation and intricate surface detail capture; scale calibration using reference objects of known absolute physical dimensions or measurement scales, which was established by pre-defined reference targets; and the final generation of a triangular mesh to produce an authentic 3D representation of the pavement texture, followed by the mapping of high-resolution texture mapping constructed based on the dense point cloud.

### 2.2. Specimen Preparation and Texture Reconstruction

In this study, four representative asphalt mixtures—AC-13, AC-16, SMA-16, and OGFC-16—were selected, the specific gradations detailed in Table 1. Basalt was utilized as the aggregate, while the binder consisted of SBS-modified asphalt (PG 76-22). The specimens were fabricated using the Superpave Gyratory Compactor (SGC) method to initially form cylinders with a diameter of 150 mm and a height of 50 mm. The bitumen content by mass of aggregate determined were 4.9% for AC-13, 4.7% for AC-16, 5.7% for SMA-16, and 4.7% for OGFC-16. Subsequently, these cylinders were precision-cut into 80 mm × 80 mm × 50 mm samples to facilitate subsequent texture measurement and analysis, and 3 specimens were fabricated for every gradation.

**Figure 2:**
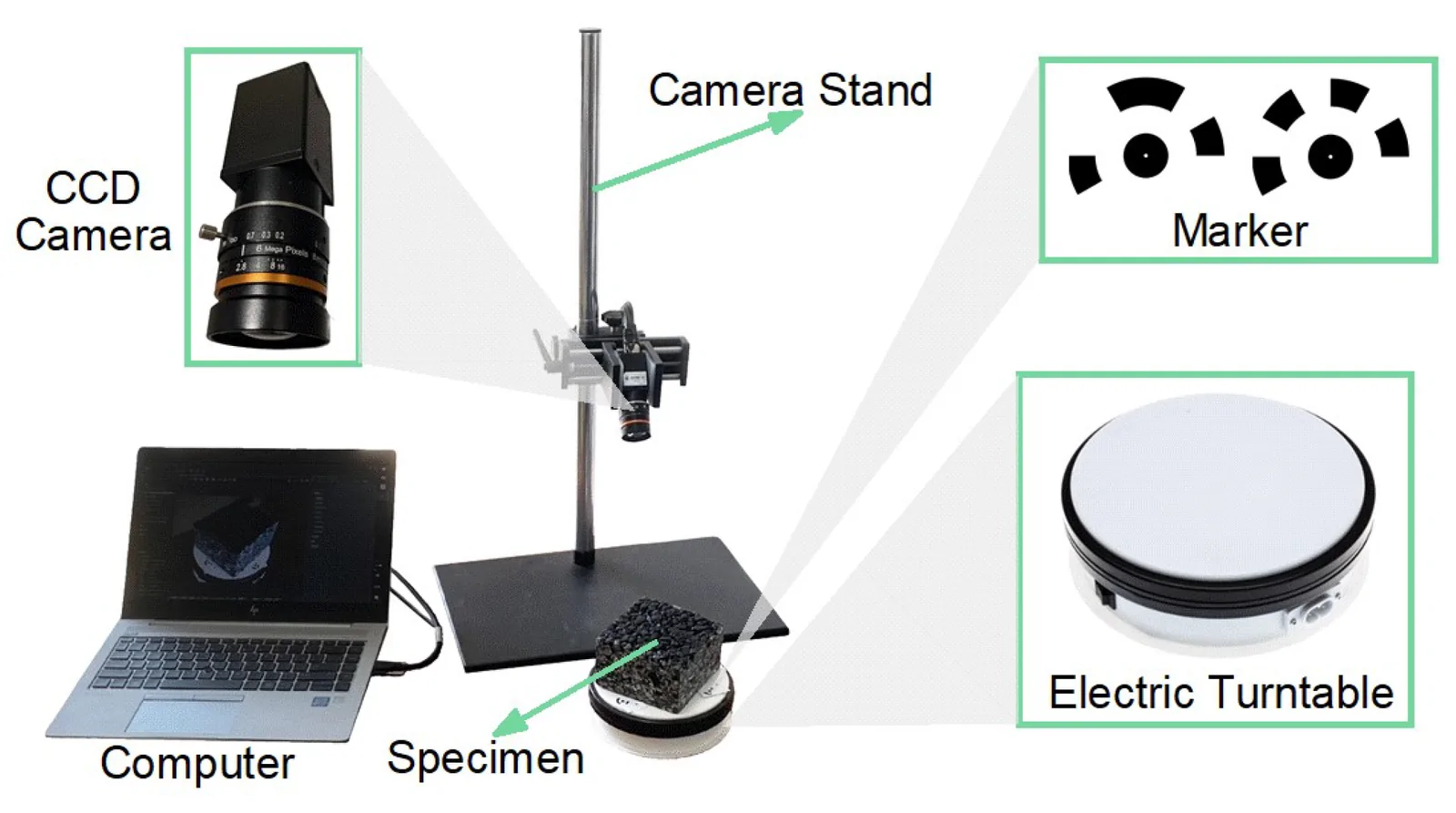
Photogrammetric system for specimen surface image acquisition.

The pavement surface texture data was acquired using the close-range photogrammetry system illustrated in Figure 2. Video sequences of each specimen surface were recorded using a Hikrobot MV-CS060-10GC industrial camera (Hangzhou Hikrobot Co., Ltd., Hangzhou, China) with a 50 mm lens, mounted on a camera stand at a fixed camera-to-surface distance of 300 mm. The video was captured at a resolution of 3072 × 2048 pixels, with the camera settings fixed at an exposure time of 20 ms. During the image acquisition process, the electric rotary stage that rotated at a constant speed of 360° per 16 s in the horizontal plane, while the camera continuously captured frames at 9.1 fps, yielding approximately 145 frames per revolution with an angular interval of approximately 2.48° between consecutive frames. The acquisition was conducted under natural lighting conditions with the angle between the camera lens axis and the specimen surface was fixed at 45°. A C-Mount lens with an adjustable range of F2.8–F16 was mounted on the camera. The aperture was manually adjusted prior to data acquisition to achieve appropriate exposure under natural lighting conditions.

In this study, an initial complete sequence of 145 photographs was captured. To systematically evaluate the impact of image quantity on reconstruction accuracy, the image sequence was downsampled into nine subsets using an equiangular sampling method. The number of extracted photographs was 145, 73, 49, 37, 29, 25, 21, 19, and 17, corresponding to angular intervals of 2.48°, 4.93°, 7.35°, 9.73°, 12.41°, 14.4°, 17.14°, 18.94°, and 21.18°, respectively. It was observed that when the number of photographs was fewer than 17, the images failed to register for successful 3D modeling. Therefore, 17 was selected as the minimum image count for this study.

## 3. Quality Evaluation Methods for Photogrammetric Texture Reconstruction

### 3.1. Acquisition of Reference Point Cloud and Point Cloud Registration

#### 3.1.1. Reference Point Cloud

**Figure 3:**
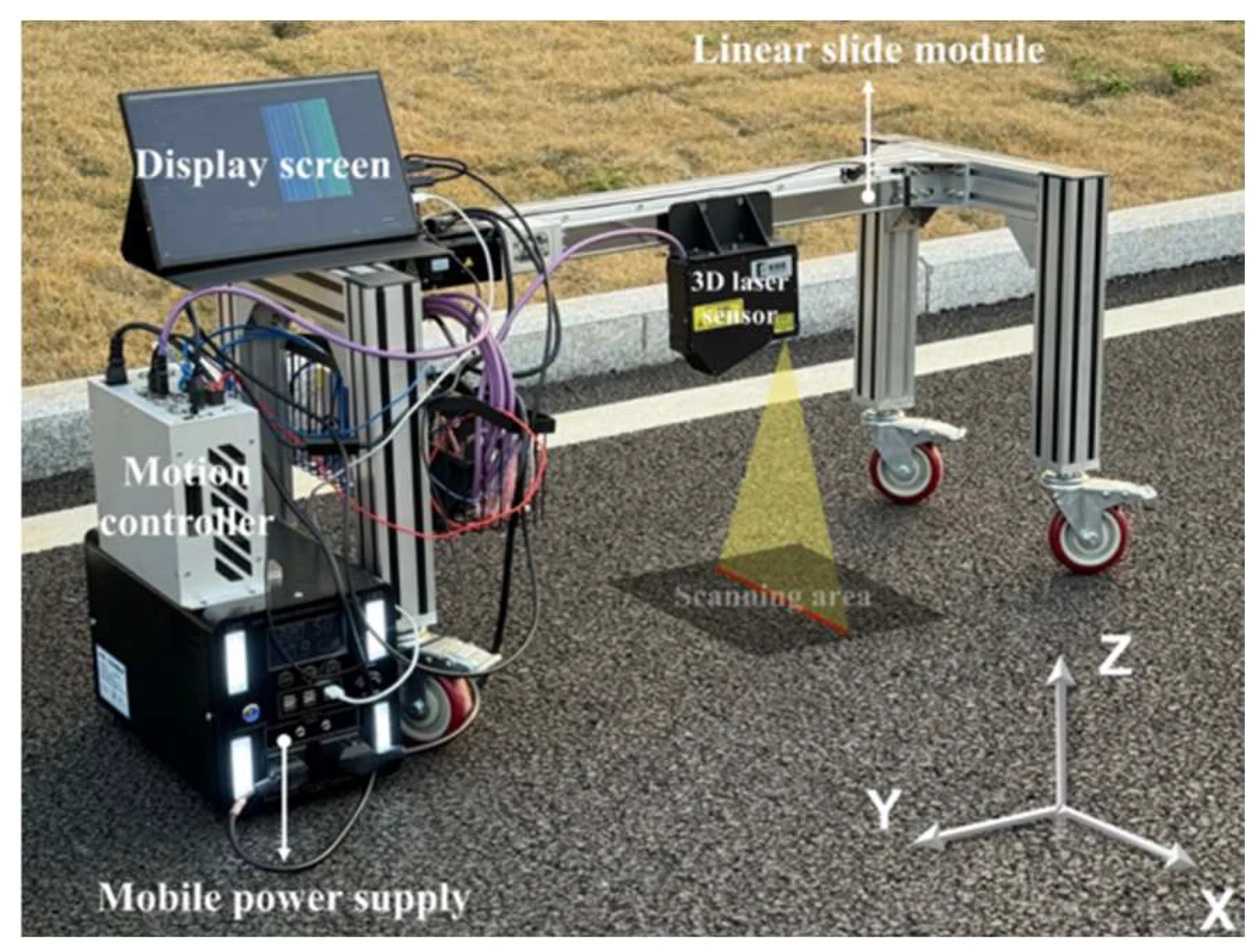
Self-developed high-precision laser scanning system.

The acquisition of the reference point cloud data was conducted using a self-developed high-precision laser scanning system (Figure 3). The system primarily consists of a linear laser sensor with accuracy of 0.02 mm (KEYENCE LJ-V7080, KEYENCE CORPORATION, Osaka, Japan), a precision displacement stage with a travel distance of 200 mm and positioning accuracy of 0.005 mm, and a data acquisition unit. The measurement accuracy of the system was factory-calibrated by the manufacturer using standard reference artifacts of known dimensions, and the measurement uncertainty was evaluated through redundant testing. The scanning parameters were configured with a scanning speed of 10 mm/s, a calibrated lateral resolution of 0.1 mm, a longitudinal resolution of 0.05 mm, and a scanning range of 120 × 120 mm. Approximately 2.4 million data points were obtained for each scanned specimen. Regarding data preprocessing, the raw laser point clouds were first regularized and leveled to ensure that the average elevation of all point clouds was zero, thereby establishing a unified reference plane for subsequent comparative analysis with the photogrammetric data. It is worth noting that mobile robotic line-laser systems such as RoboTex have been previously developed for field pavement texture measurement.

#### 3.1.2. Point Cloud Registration

**Figure 4:**
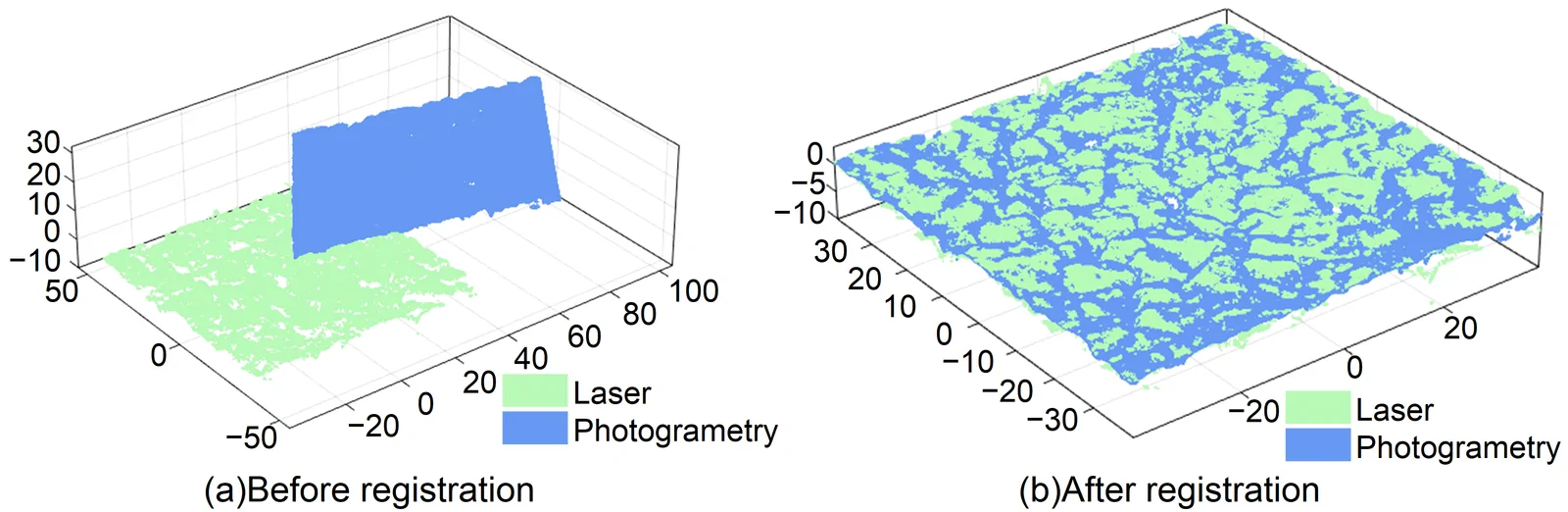
Before and after the point data registration.

Elevation deviation is the core indicator for evaluating the accuracy of photogrammetric 3D reconstruction, quantifying the vertical geometric differences between the reconstructed point cloud and the laser-scanned reference point cloud. Since the two acquisition systems (laser scanning and photogrammetry) operate within independent local coordinate systems, discrepancies exist in the initial positions and orientations of the captured point cloud data (Figure 4a). To eliminate systematic positional bias and facilitate elevation deviation calculation, the texture data obtained from different modeling methods must be aligned to a unified spatial reference. In this study, the Iterative Closest Point (ICP) algorithm was employed to perform precise point cloud alignment by iteratively optimizing the search for corresponding points and calculating the rigid body transformation matrix; the maximum number of iterations was set to 50, and the convergence criterion was defined as a transformation difference below 10^−6^. Upon convergence, the registration root-mean-square (RMS) error was consistently below 1.5 mm for all specimens. The aligned point clouds are shown in Figure 4b.

### 3.2. Evaluation Indicators for Texture Reconstruction Quality

#### 3.2.1. Density of Point Data

Point cloud density is a descriptive metric to characterize the sampling intensity of 3D reconstruction, as it directly reflects the sampling intensity of the reconstructed model within the spatial domain and its capability to represent the detailed surface features of the specimen. Thus, this study employs the number of points per unit area for the quantitative statistical analysis of point cloud density.

#### 3.2.2. Point Cloud Deviation

It should be noted that point cloud density alone does not serve as a direct indicator of reconstruction accuracy, since a higher density does not necessarily imply higher geometric fidelity. Thus, after completing the registration between the point clouds obtained from the photogrammetric method and the laser scanner, the deviation (DEV) and absolute deviation (ADEV) between these two methods are calculated according to Equations (1) and (2).

(1) $$DEV_{Z}\left(x,y\right)=Z_{\mathrm{Photogrammetry}}\left(x,y\right)-Z_{\mathrm{Laser}}\left(x,y\right)$$

(2) $$ADEV_{Z}\left(x,y\right)=\left|Z_{\mathrm{Photogrammetry}}\left(x,y\right)-Z_{\mathrm{Laser}}\left(x,y\right)\right|$$

where (x, y) represents the planar coordinates of a surface point, while Z_photogrammetry_ (x, y) and Z_laser_ (x, y) denote the surface elevations at the position (x, y) obtained via photogrammetry and laser scanning, respectively.

#### 3.2.3. Texture Parameters Deviation

The selected surface texture parameters include: (a) Root mean square height (Sq), which quantitatively describes the degree of surface topographical fluctuation of each point relative to the mean plane; (b) Skewness (Ssk), which reflects the degree of asymmetry in the surface height distribution relative to the mean plane; (c) Kurtosis (Sku), which reflects the sharpness of the texture, with higher values indicating greater sharpness; (d) Root mean square slope (Sdq), which quantitatively describes the degree of local slope variation on the surface. The above pavement texture parameters can be calculated according to Equations (3)–(6).

(3) $$S_{q}=\sqrt{\frac{1}{A}\iint_{A}Z^{2}(x,y)\;dx\;dy}$$

(4) $$S_{\mathrm{sk}}=\frac{1}{{S_{q}}^{3}}\left[\frac{1}{A}\iint_{A}Z^{3}(x,y)dxdy\right]$$

(5) $$S_{ku}=\frac{1}{S_{q}^{4}}\frac{1}{A}\iint_{A}Z^{4}(x,y)dxdy$$

(6) $$S_{dq}=\sqrt{\frac{1}{A}\iint_{A}\left[{\left(\frac{\partial z(x,y)}{\partial x}\right)}^{2}+{\left(\frac{\partial z(x,y)}{\partial y}\right)}^{2}\right]\;dx\;dy}$$

where Z(x, y) represents the elevation of the surface at coordinates (x, y) relative to the mean plane elevation, A is the area of the measurement region, the ∂z/∂x and ∂z/∂y are the partial derivatives of the surface in the x and y directions, respectively.

## 4. Results and Discussion

### 4.1. Point Cloud Density Comparison

**Figure 5:**
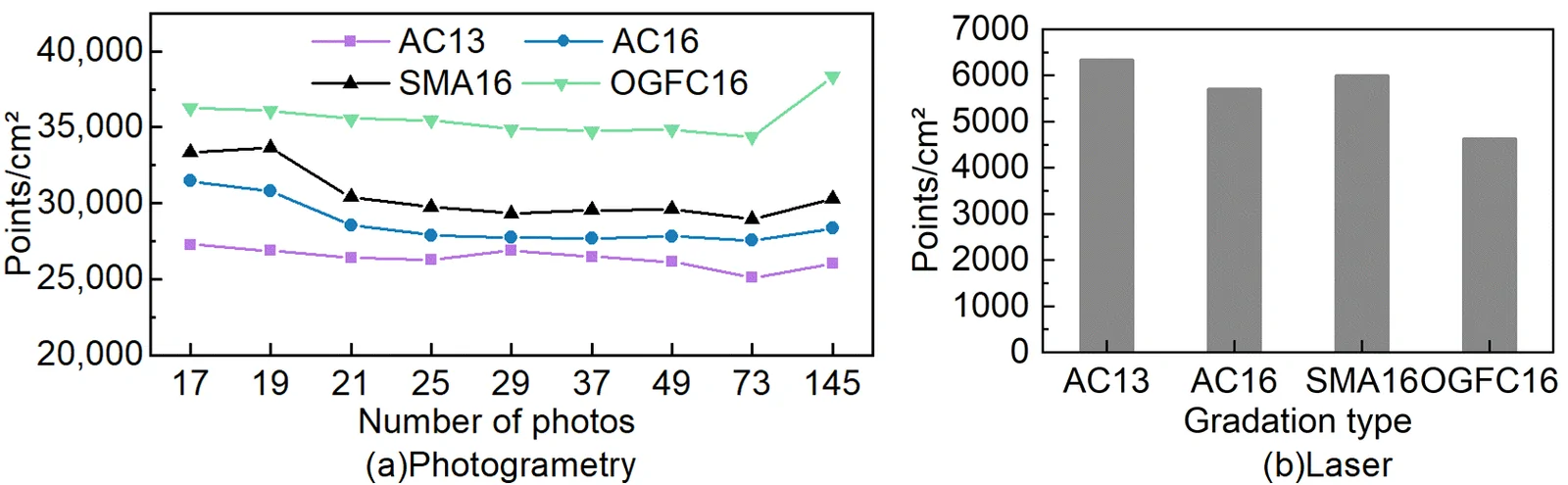
Point cloud density of two methods (points/cm^2^).

Figure 5 illustrates the comparison of point cloud densities between the photogrammetric reconstruction models—under four different gradations and varying photograph quantities—and the results obtained from laser scanning. According to Figure 5a, the point cloud density for all gradations is significantly higher than that of the laser scanning results, stabilizing at 25,000 to 38,000 points/cm^2^. Specifically, OGFC-16 exhibits the highest point cloud density, reaching approximately 35,000 points/cm^2^, since its numerous surface voids and high degree of exposed aggregate provide an abundance of image feature points, such as angularities and pore boundaries, which greatly enhances the success rate of pixel-level matching. SMA-16 ranks second in density as the surface texture formed by aggregate interlocking provides a substantial number of feature points. Notably, the point cloud density of AC-16 is generally higher than that of AC-13. This is attributed to the larger nominal maximum aggregate size of AC-16, which results in a slightly higher macroscopic surface roughness compared to AC-13, thereby generating more light-dark variations and textural details in the images.

Further observation of Figure 5a reveals that the point cloud density can be qualitatively divided into three evolutionary stages as the number of photographs increases based on visual inspection of the trend. The first stage appears as the distorted mutation period (17 to 21 photos). Limited by the small number of photographs and insufficient overlap, the accuracy of multi-view geometric intersection becomes unreliable or even fails. This leads to severe deviations in depth map estimation and generates significant spatial high-frequency noise, resulting in a phenomenon of counter-intuitive high density due to mismatches followed by a rapid decline. This is followed by the stable plateau period (21 to 73 photos). Within this broad range, the point cloud density curves for all pavement gradations tend to level off, indicating that the image overlap rate is maintained within a reasonable threshold, photogrammetry demonstrates excellent robustness and stable data output. Finally, the sequence enters the growth period (73 to 145 photos). An ample number of photographs ensures a high level of overlap, enabling denser multi-view geometric matching, which leads to a clear upward trend in point cloud as the number of photographs increases.

In contrast, Figure 5b shows that the point cloud density for laser scanning is within a much lower range of 4500 to 6500 points/cm^2^. The density ranking of different gradations follows the order of AC-13 > SMA-16 > AC-16 > OGFC-16, presenting a reversed trend from which obtained by close-range photogrammetry. This phenomenon is attributed to structures like OGFC-16, which possess deep and large pores; the laser beam is susceptible to occlusion or multi-path reflections when illuminating edges or deep holes, leading to a reduction in effective return points. Comparing the two technologies, the point cloud density of laser scanning models is approximately 1/8 to 1/4 of that obtained through photogrammetry. This discrepancy stems primarily from the different modeling mechanisms: photogrammetry relies on a pixel-based dense matching mechanism, where dense matching algorithms (such as MVS) can perform depth calculations at the pixel level, theoretically generating 3D coordinate points for every visible pixel. Especially on surfaces like OGFC-16 with rich macroscopic textures, the abundance of feature points and high-contrast information in the images provides a fertile data source for the algorithm, achieving extremely fine sampling coverage. Conversely, as an active measurement technology, the point cloud density of laser scanning is strictly limited by hardware pulse repetition frequency, scanning speed, and the physical spacing of the laser beams, making it unable to achieve pixel-level point cloud densification by increasing image resolution as photogrammetry does.

### 4.2. Point Cloud Elevation Deviation

**Figure 6:**
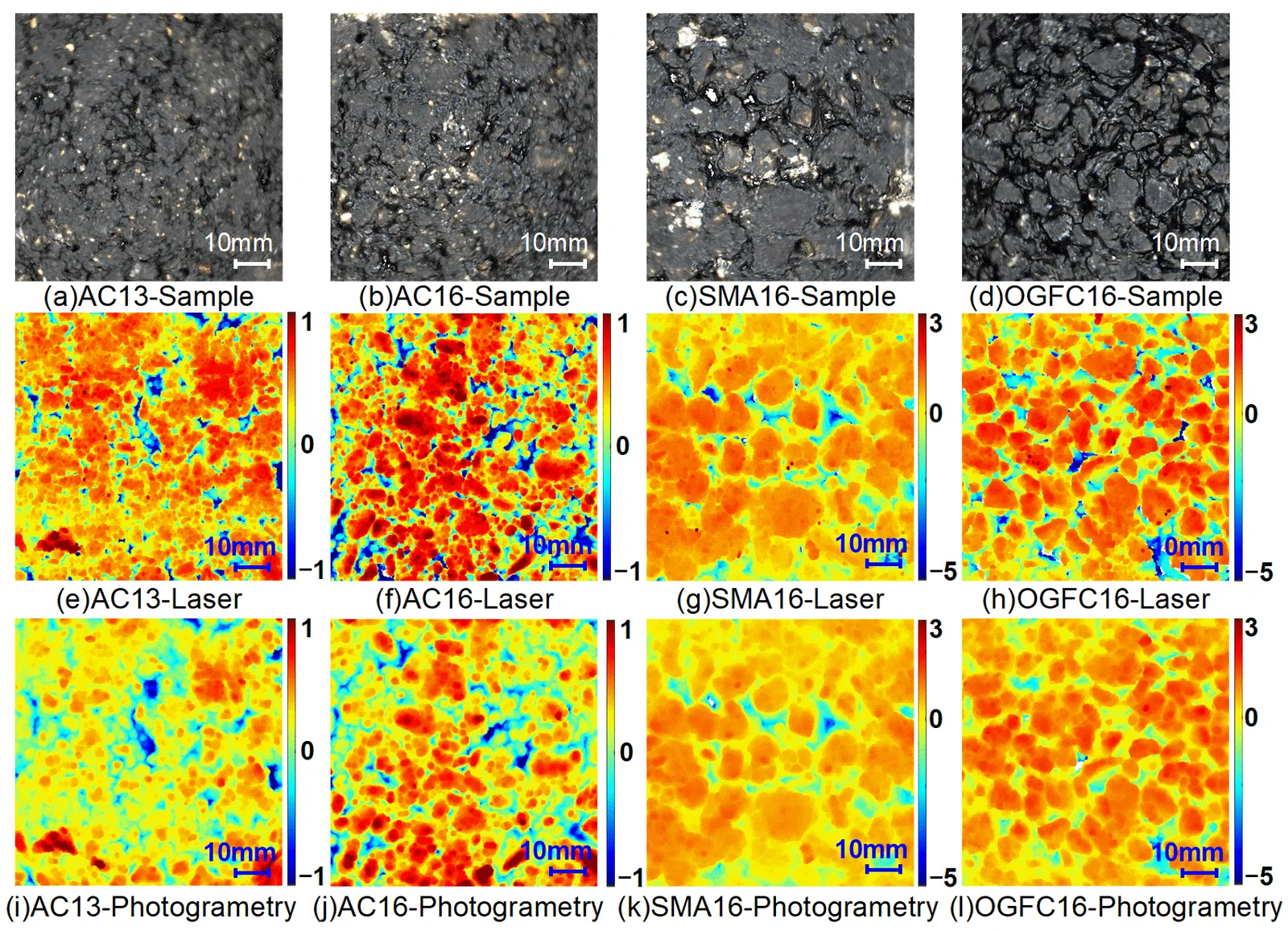
Original images and reconstruction results of asphalt mixtures via laser scanning and photogrammetry.

Figure 6 illustrates the surface texture characteristics of AC-13, AC-16, SMA-16, and OGFC-16, captured using photogrammetry and laser scanning respectively. Specifically, (a)–(d) are the original RGB images of the specimen surfaces; (e)–(h) are the texture models reconstructed from the 3D point cloud data obtained by the high-precision laser scanner; and (i)–(l) are the texture models reconstructed via photogrammetry under the condition of the maximum number of images (145 photos).

By comparing the original RGB images in Figure 6a–d with the corresponding 3D models, it can be observed that both the models based on high-precision laser scanning (Figure 6e–h) and the photogrammetric models based on 145 images (Figure 6i–l) highly accurately restore the spatial distribution, geometric contours, and void characteristics of the pavement texture. This validates the reliability of both modeling methods in capturing the 3D morphology of asphalt mixtures. However, there are noticeable differences in the resolution of texture details. The laser-scanned models exhibit sharper edges and richer surface fluctuations, enabling the clear replication of fine textures on the pavement surface. In contrast, while the photogrammetric models are highly consistent with the former at a macroscopic scale, they display a certain degree of smoothing in micro-detail processing, showing a slightly inferior capability for capturing small-scale textures compared to laser scanning.

**Figure 7:**
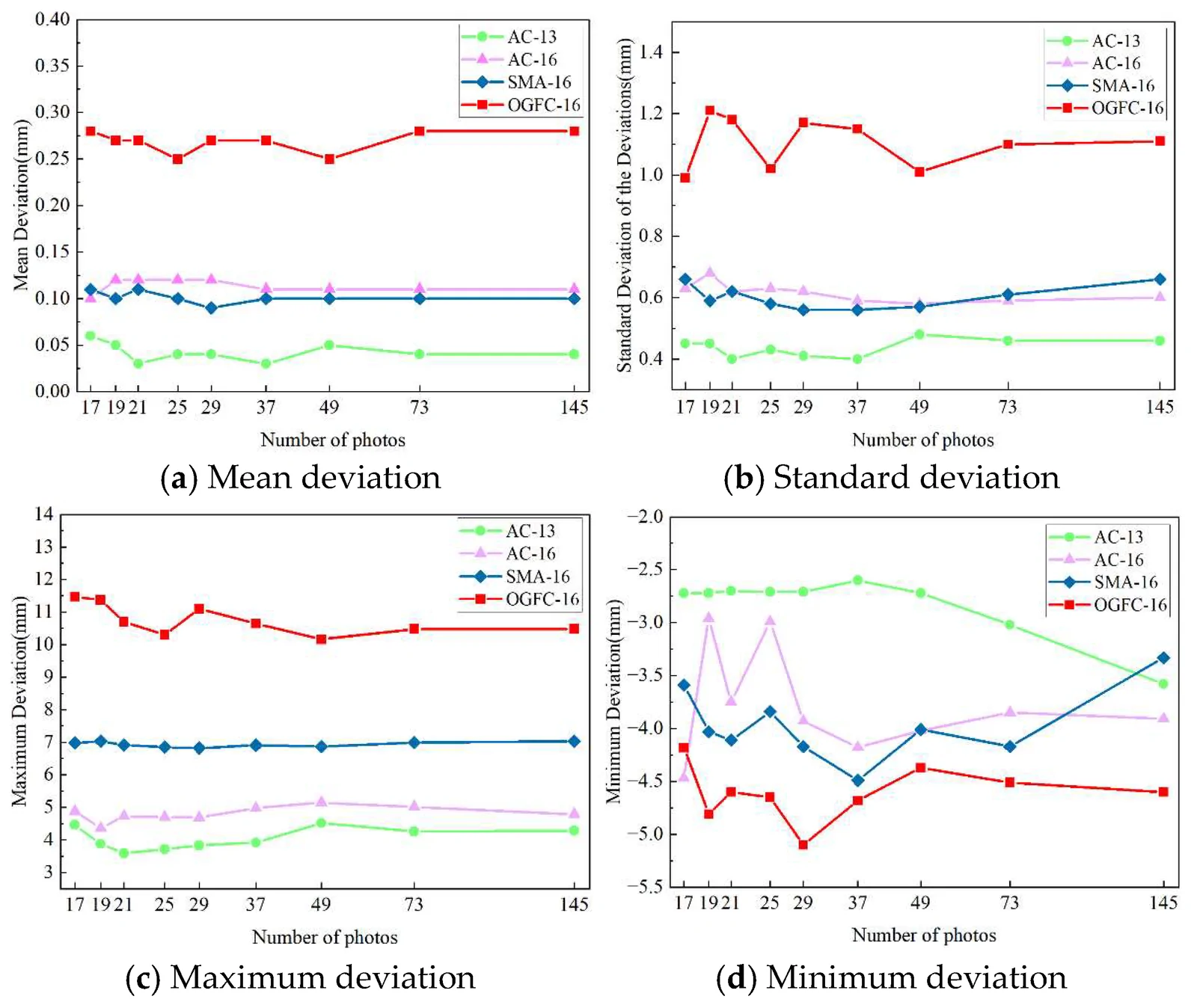
Deviations for different gradation types under different photograph quantities.

To facilitate a detailed comparison of the differences between the modeling methods, the models obtained via laser scanning were used as the benchmark. Point cloud registration was performed, and the deviation (DEV) between point clouds was calculated using Equation (1). Figure 7a–d provide a detailed breakdown of the statistical characteristics of the deviations for the four gradation types under different photograph quantities, including the mean deviation, standard deviation, maximum deviation, and minimum deviation.

A comparison of Figure 7a–d indicates a significant correlation between deviation-related indicators and the mixture gradation type. For instance, the mean deviation of AC-13 remains within the range of 0.03–0.06 mm, indicating no significant systematic offset and demonstrating excellent reconstruction precision. However, as the gradation coarsens, the standard deviation increases to 0.56–0.68 mm for AC-16 and SMA-16, eventually jumping to 0.99–1.21 mm for OGFC-16. This stepwise increase is also reflected in the maximum deviation, which rises from 3.59 mm for AC-13 to over 11.0 mm for OGFC-16, while the minimum deviation remains relatively concentrated across all gradations between −2.6 mm and −5.1 mm.

Interestingly, although the OGFC point cloud density is 25% higher than that of AC-13, its mean deviation is also the highest. This correlation implies that for open gradations, the increased density may be predominantly composed of invalid or low-precision points rather than true morphological data. A plausible explanation is that photogrammetry achieves robust multi-view matching on dense-graded surfaces with high flatness and continuous feature points. Conversely, for surfaces like OGFC with complex deep-pore structures, structural occlusion and shadow interference often lead to local matching failures, thereby increasing the overall deviation level. Consequently, coarser gradations significantly amplify modeling errors.

Observing the performance across the range of 17 to 145 photographs, both the mean deviation and standard deviation exhibit no obvious directional trend after the initial fluctuations, based on visual inspection of the curves (Figure 7). For example, the mean deviation for SMA-16 remains stable at approximately 0.10 mm even as the number of photos increases nearly eightfold. This observation is qualitatively consistent with the preceding point cloud density analysis, and tentatively suggests that once image overlap reaches the robust trigger threshold for the algorithm, merely increasing image redundancy yields at best marginal improvements in statistical modeling accuracy.

Notably, for gradations like AC-13, the maximum deviation exhibits a slight upward trend at higher photo counts. A plausible explanation for this observation is that the increased point cloud density captures finer surface burrs and void edges, which often contain lighting dead zones that may potentially trigger high-frequency matching noise. Furthermore, the absolute value of the maximum positive deviation is generally higher than that of the minimum negative deviation, reflecting an apparent asymmetric error characteristic in photogrammetric pavement modeling. In the case of OGFC-16, the maximum positive deviation is approximately 2.2 times the absolute value of the maximum negative deviation. These extreme positive values are tentatively attributed to spatial high-frequency noise and floating artifacts above aggregate edges or complex voids. In contrast, negative deviations are physically constrained by the actual bottom of the pavement pores. Due to limited viewing angles, the downward reach of the point cloud tends to converge with the effective detection depth of the laser scanner, resulting in a smaller fluctuation range.

### 4.3. Point Cloud Elevation Deviation Distribution

#### 4.3.1. Distribution of the Absolute Elevation Deviation

**Figure 8:**
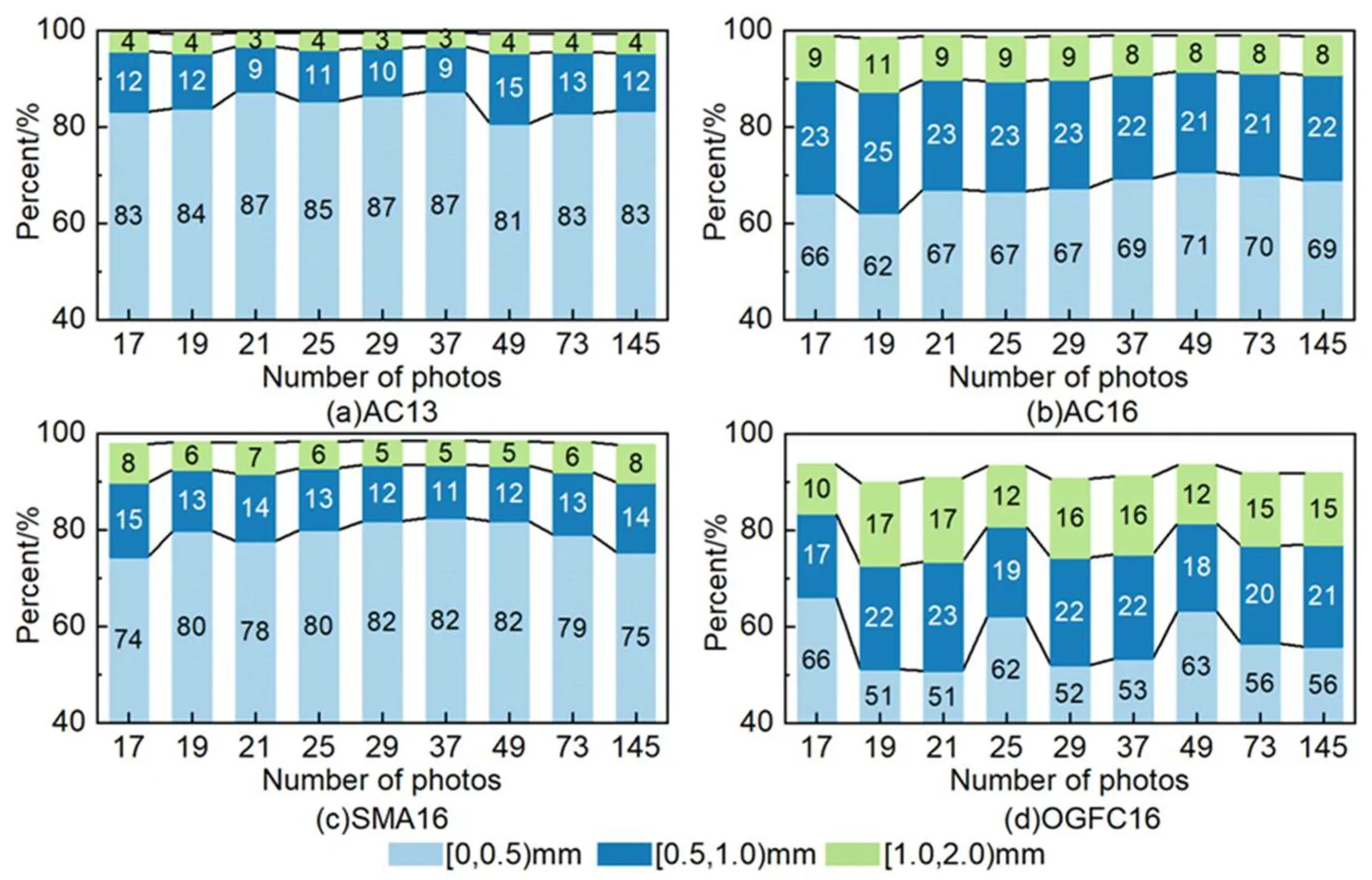
Distribution of the absolute elevation deviation of the point cloud.

Figure 8 illustrates the distribution of the absolute elevation deviation (ADEV) of the point cloud for different gradation types under varying quantities of photographs.

Based on the observed ranges, AC-13 exhibits the highest reconstruction fidelity, with the proportion of point clouds in the 0–0.5 mm precision interval reaching as high as 81% to 87%, compared with 62–71% for AC-16 and 74–82% for SMA-16. It is noteworthy that while SMA-16 has an aggregate size similar to AC-16, its proportion in the 0–0.5 mm interval (74% to 82%) is significantly superior to that of AC-16. This suggests a potential advantage for SMA-16 over AC-16 despite their similar nominal aggregate sizes, possibly due to SMA-16’s distinctive interlocking texture, which may facilitate feature matching.

Conversely, OGFC-16, characterized by a complex structure with large voids, is severely affected by multi-view geometric occlusion. Consequently, its proportion of high-precision points reaches a minimum of only 51%, while the share of high-deviation points in the 1.0–2.0 mm range increases markedly. Although OGFC-16 has the lowest proportion of points with an elevation deviation within 0.5 mm, it possesses the highest point cloud density (Figure 5), which confirms the presence of substantial noise data. Overall, the reconstruction accuracy of AC-13 and AC-16 mixtures is significantly better than that of SMA-16 and OGFC-16.

Within the plateau period (21 to 73 photos), the proportions of the deviation intervals for each gradation remain remarkably stable. For instance, the standard deviation for SMA-16 consistently stays between 0.56 mm and 0.62 mm, which validates the strong statistical robustness of photogrammetry once the overlap threshold is met. However, upon entering the growth period (up to 145 photos), the proportion of points in the high-precision intervals for AC-13 and SMA-16 actually experiences a slight decline compared to the mid-stage, dropping to 83% and 75%, respectively. The substantial increment in point cloud density suggests that ultra-high-density sampling captures more complex edges; while this improves resolution, it simultaneously introduces more high-frequency noise caused by lighting shadows. This reflects a trade-off in modeling between point density and noise reduction. Therefore, the accuracy does not significantly improve with an ever-increasing number of photographs. To achieve an optimal balance between point cloud density and precision, a range of 21 to 49 photographs is recommended as the appropriate quantity.

#### 4.3.2. Distribution of the Spatial Elevation Deviation

**Figure 9:**
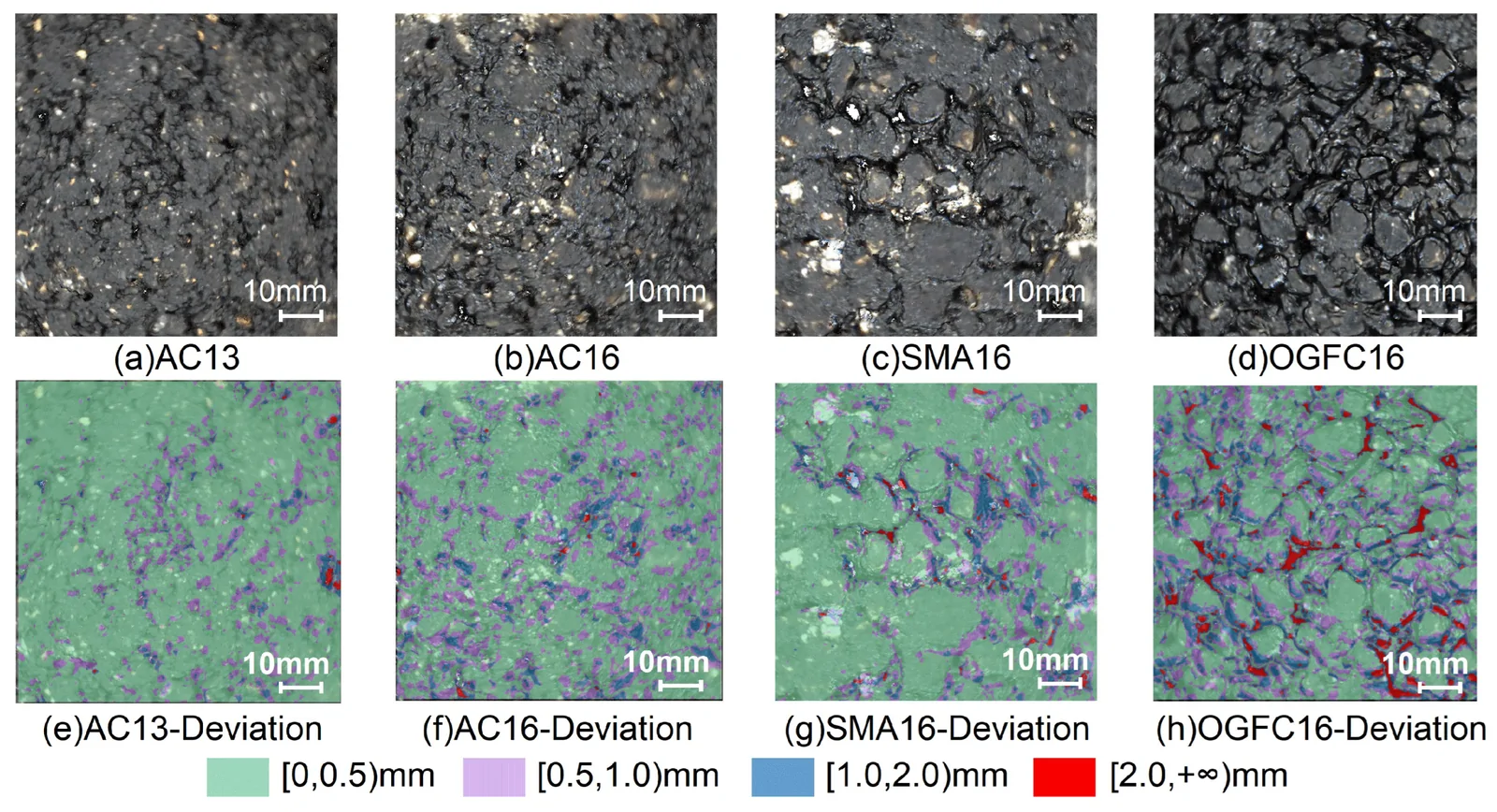
Distribution of absolute deviation on the specimen surface.

To intuitively analyze the spatial distribution of the deviations, this study generated spatial distribution maps of the absolute elevation deviations (ADEV) for the four types of pavement gradations reconstructed using 145 photographs, as shown in Figure 9e–h.

The combined visual and quantitative analysis (Figure 8) reveals the following patterns. In the relatively flat regions at the top of the aggregates, all four gradations are predominantly covered by the green color representing high precision [0, 0.5) mm. Among them, AC-13 exhibits the highest proportion in this high-precision class (83%), followed by SMA-16 (75%), AC-16 (69%), and OGFC-16 (56%), confirming the excellent reconstruction fidelity of photogrammetry for convex surfaces.

In contrast, areas with larger deviations (>1.0 mm), are primarily concentrated at aggregate edges, interlocking gaps, and deep pores. Specifically, for AC-13 and AC-16, the elevated deviation points are scattered at the junctions of aggregates, with the ≥1.0 mm class occupying only 4.6% and 9.2% of the total area, respectively. For SMA-16, the deviation points exhibit a more pronounced network distribution following the gaps between aggregates, with the ≥1.0 mm class covering 10.1% of the surface. By comparison, OGFC-16 shows a substantial increase in the highest-deviation class (≥2.0 mm, red), which accounts for as much as 23% of the entire mapped area, forming dense red patches at the locations of deep pore structures.

This quantified spatial distribution not only directly corroborates the earlier finding that OGFC-16 exhibits the largest maximum deviation values, but also provides a statistical basis for attributing local modeling failures to multi-view geometric occlusion caused by complex pore structures. The area proportion of the ≥2.0 mm deviation class serves as a quantitative indicator of the extent of photogrammetric degradation, with OGFC-16 showing a value approximately 12 times that of AC-13.

### 4.4. Indicator Deviation

**Figure 10:**
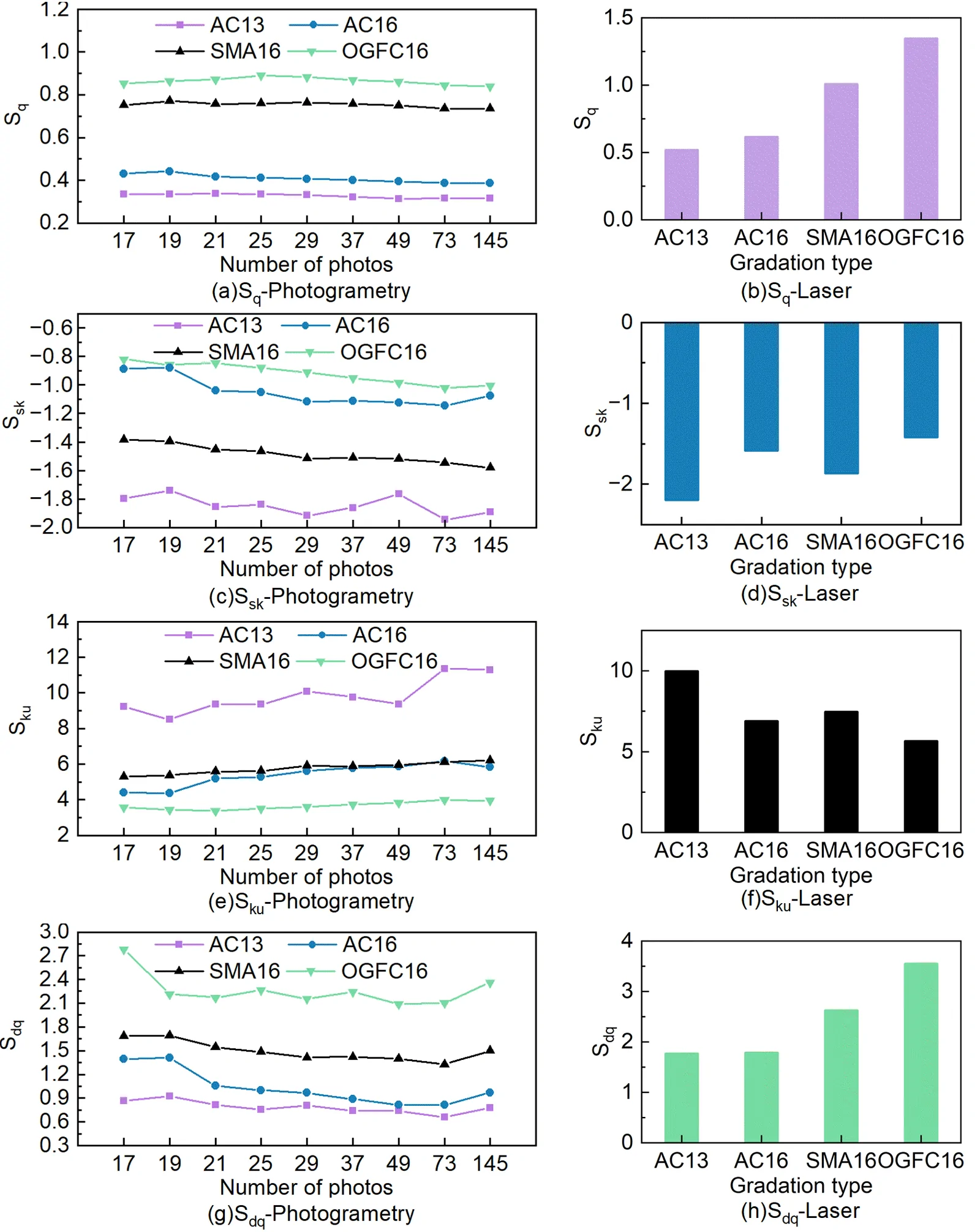
Influence of photo number and gradation on texture indicators.

Figure 10 presents the root mean square height (Sq), skewness (Ssk), kurtosis (Sku), and root mean square slope (Sdq) calculated for different gradations using photogrammetry with varying numbers of photographs, as well as those obtained from laser scanner.

As shown in Figure 10a, the ranking for the root mean square height Sq indicator of four types of gradations remains consistent across both modeling methods: OGFC-16 > SMA-16 > AC-16 > AC-13. Although in terms of numerical magnitude, the photogrammetric results are systematically lower than the laser scanning reference values. Taking AC-13 as an example, the laser scanning (Figure 10b) gives an Sq of approximately 0.5 mm, while the photogrammetric value (Figure 10a) stabilizes around 0.35 mm, which is only 70% of the laser reference. Similarly, for OGFC-16, the laser scanning value is about 1.35 mm, and the photogrammetric value is about 0.85 mm, the ratio being approximately 0.63. This indicates that photogrammetry exhibits a “smoothing effect” when reconstructing the microscopic texture depth of pavement surfaces, resulting in systematically underestimated height parameters. However, photogrammetry can perfectly reproduce the relative ranking of roughness among different pavement gradations. Even though the absolute values from photogrammetry are generally lower than those from laser scanning, its ability to distinguish roughness among different gradations remains strong. Furthermore, the Sq values obtained via photogrammetry do not show significant fluctuations as the number of photographs increases. This indicates that Sq, as a second-order statistical moment, is highly robust to variations in noise and sampling density. Therefore, when using this parameter, 17–19 photographs are sufficient, and there is no need to excessively increase the number of photographs in pursuit of higher precision.

A comparison between Figure 10c and Figure 10d indicates that photogrammetry can reasonably reproduce the skewness characteristics of the surface. For instance, the laser scanning value for AC-13 is approximately −2.2, while the photogrammetric stable value ranges from about −1.9 to −2.0, yielding a ratio of approximately 0.9. For OGFC-16, the laser reference is about −1.4, and the photogrammetric value stabilises around −1.0 to −1.1, corresponding to a ratio of approximately 0.75–0.8. Regarding the influence of the number of photographs, Figure 10c shows that the curves for the coarse-graded mixtures remain relatively stable, whereas the fine-graded mixture AC-13 exhibits considerable fluctuation. This phenomenon lies in Ssk, as a third-order statistical moment, has a magnification effect on surface point-cloud noise. On finely textured, highly repetitive surfaces such as AC-13, the photogrammetric feature-matching algorithm struggles to find sufficiently abundant. Increasing the number of photographs can enhance point-cloud density and thereby suppress the influence of random noise on the statistical distribution, which gradually drives the results to converge towards the true reference values.

Crucially, these findings do not contradict our earlier conclusion regarding mean deviation. Instead, they reveal a complementary diagnostic insight: a low mean deviation does not guarantee a low Ssk. For dense-graded, smooth pavements, photogrammetry achieves excellent overall fidelity but suffers from sporadic, high-magnitude mismatches due to texture ambiguity. For open-graded OGFC, the error is more broadly distributed across pore structures but remains physically bounded by the pore depth, thus not necessarily producing the same level of extreme skewness.

Similarly, for the fourth-order statistical moment Sku, even a very small number of extreme noise points can cause numerical fluctuations. As shown in Figure 10f, the laser scanning Sku value for AC-13 is approximately 10, whereas the photogrammetric values in Figure 10e rise to 11–12 as the number of photographs increases, corresponding to a ratio of approximately 1.1–1.2. For SMA-16, the laser reference is about 7.5, while the photogrammetric value is approximately 6, yielding a ratio of about 0.8. Nevertheless, the degree of surface extremity calculated from photogrammetric data is even higher than that from laser scanning. A plausible explanation is that, on the finely textured and highly repetitive AC-13 surface, photogrammetric matching noise is mistakenly identified as high-frequency extreme texture features. Furthermore, the AC-13 curve in Figure 10e shows a clear upward trend, indicating that as the number of photographs increases, the photogrammetric algorithm captures an increasing number of anomalous extreme points, which progressively elevates the computed kurtosis. This implies that when using photogrammetry to evaluate Sku, increasing the number of photographs may introduce instability; thus, one should be cautious that an excessively high kurtosis value may reflect algorithmic artifacts rather than genuine pavement surface characteristics. Figure 10g,h reveal that photogrammetric Sdq values are generally lower than those from laser scanning. For example, the laser scanning Sdq value for AC-13 is approximately 1.8, whereas the photogrammetric value stabilises around 0.8–0.9 (about 0.5 times). For OGFC-16, the laser reference is about 3.5, while the photogrammetric value is approximately 2.4 (about 0.7 times). This indicates that the surface reconstructed via photogrammetry appears smoother than the real surface, with some microscopic slope details being lost. Regarding the influence of the number of photographs, Figure 10g shows that Sdq exhibits a slight downward trend as the number of photographs increases (particularly for OGFC-16). A plausible explanation is that additional photographs introduce more viewing constraints, which encourages the reconstruction algorithm to generate smoother and topologically simpler surfaces, thereby further flattening subtle texture undulations and leading to a modest reduction in the computed root-mean-square gradient.

## 5. Conclusions

This systematic study investigated the factors influencing pavement texture reconstruction using close-range photogrammetry, with high-precision laser scanning serving as the reference benchmark. The reconstruction accuracy was evaluated across three dimensions—point cloud density, elevation deviation, and 3D texture indicators—leading to the following primary conclusions:

• Dense-graded mixtures (AC-13, AC-16, SMA-16) consistently outperform OGFC-16 in all deviation metrics and high-precision point proportions. The complex pores of OGFC cause occlusion and matching failures, confirmed by spatial deviation maps where red (≥2.0 mm) areas concentrate over deep voids. For dense-graded, 17–21 photos achieve mean deviation 0.3–0.5 mm; for OGFC, 20–25 photos are needed but precision remains lower. Additional photos yield marginal gains.
• Texture parameters correlate with laser scanning but require gradation-specific interpretation. The Sq and Sdq are systematically underestimated (smoothing effect), yet reliably rank gradations. The Ssk and Sku are sensitive to outliers, on AC-13, and higher photo counts may increase noise rather than true sharpness.

While the above findings provide valuable quantitative insights, several limitations of the present work should be acknowledged. First, this study covered four gradation types, each represented by three slab specimens. Although these are typical of Chinese pavement practice, the results cannot be generalised to other aggregate types, nominal maximum sizes, or surface textures without additional testing. Second, all reconstructions were performed using fixed camera–object geometry and uniform laboratory lighting. The observed trends may not hold for other parameter settings, or outdoor illumination conditions. Finally, this study was confined to laboratory-cured, dry specimens under static viewing conditions, while field pavements are subject to moisture, contamination, and variable lighting, all of which may affect reconstruction fidelity. In light of these limitations, future research should expand the experimental matrix to include a wider variety of pavement materials and gradations, compare multiple reconstruction algorithms and parameter settings, conduct field-based validation under realistic conditions. Overall, the present work provides a systematic baseline and demonstrates the critical role of gradation and reconstruction settings in dictating photogrammetric performance.

## Figures and Tables

**Table 1:** Gradation of asphalt mixture.

Gradation Type		Sieve Size (mm)										
		19	16	13.2	9.5	4.75	2.36	1.18	0.6	0.3	0.15	0.075
Passing (%)	AC13	100	100	95	76.5	53	37	26.5	19	13.5	10	6
	AC16	100	95	84	70	48	34	24.5	17.5	12.5	9.5	6
	SMA16	100	95	75	55	26	19.5	18	15	12.5	11.5	10
	OGFC16	100	95	80	57.5	21	16	12	9.5	7.5	5.5	4

## Data Availability

The original contributions presented in this study are included in the article. Further inquiries can be directed to the corresponding author.
